# The complete chloroplast genome of *Elaeagnus bambusetorum* hand.-mazz. 1933 and its implications for phylogenetic relationships in the *Elaeagnus* genus

**DOI:** 10.1080/23802359.2024.2403413

**Published:** 2024-09-12

**Authors:** Yuchen Yang, Jun Li, Zhixiang Zhang, Mengxue Zhang

**Affiliations:** a Faculty of Geography, Yunnan Normal University, Kunming, China; b Honghe Prefecture Institute of Forestry and Grassland, Mengzi, China; c School of Ecology and Nature Conservation, Beijing Forestry University, Beijing, China; d School of Communication, Yunnan Normal University, Kunming, China

**Keywords:** Chloroplast genome, *Elaeagnus bambusetorum*, phylogenetic analysis

## Abstract

*Elaeagnus bambusetorum* Hand.-Mazz. is a rare plant from China in the Elaeagnaceae family. In this study, we sequenced its complete chloroplast genome. The whole chloroplast genome was 152,265 bp in length, containing a pair of inverted repeats of 25,897 bp, separated by large single copy and small single copy regions of 82,291 bp and 18,180 bp, respectively. The complete genome contained 113 genes, including 79 protein-coding genes, 30 tRNA genes, and 4 rRNA genes. The overall GC content was 37.1%. Phylogenetic analysis using the whole chloroplast genome revealed that *E. bambusetorum* is sister to *E. loureirii* and *E. conferta*. Our study provides valuable insights into the genetic information of *E. bambusetorum*, which may have important implications for species conservation.

## Introduction

*Elaeagnus bambusetorum* (Elaeagnaceae) is an endangered deciduous tree that holds a unique and precarious place in the biodiversity of Yunnan Province, China. This rare species was first documented by the Austrian botanist Heinrich Handel-Mazzetti in 1915, during his botanical explorations. After its initial discovery, *E. bambusetorum* seemed to vanish from the botanical records, not being observed again for 106 years until its recent rediscovery in Mengzi. However, the rediscovered population is alarmingly small, with fewer than 20 individual plants recorded. These trees are predominantly found near villages, making them highly susceptible to human activities and the presence of domestic animals. This exposure not only threatens their physical well-being but also exacerbates the risk of genetic bottlenecks, further endangering the species’ survival (Nazir et al. 2020). The survival of *E. bambusetorum* is critical, not just for local biodiversity, but also for the preservation of global genetic diversity among wild plants. Understanding and conserving such rare species necessitates detailed genetic information, which can help in developing effective conservation strategies. As highlighted by research, obtaining, and analyzing genetic data is vital for the conservation of rare plant species (Kahilainen et al. 2014). The genus *Elaeagnus* consists of approximately 70-80 species. However, the taxonomy within this genus is still unresolved due to insufficient information on infraspecific morphological variations (Farzaei et al. 2015). To address this, it is crucial to infer phylogenetic relationships among Elaeagnus species, which would provide a more solid basis for taxonomic revisions.

In our study, we focused on sequencing and annotating the complete chloroplast genome of E. bambusetorum. Chloroplast genomes are invaluable for phylogenetic analyses, genetic diversity assessments, and conservation efforts. They offer rich genetic resources that help elucidate evolutionary relationships and inform conservation strategies (Dong et al. 2022; Guo et al. 2023). By leveraging the chloroplast genome data, we aimed to infer the phylogenetic relationships within the Elaeagnus genus, thus contributing to the broader understanding and conservation of this critically endangered species.

## Materials and methods

**Figure 1.**
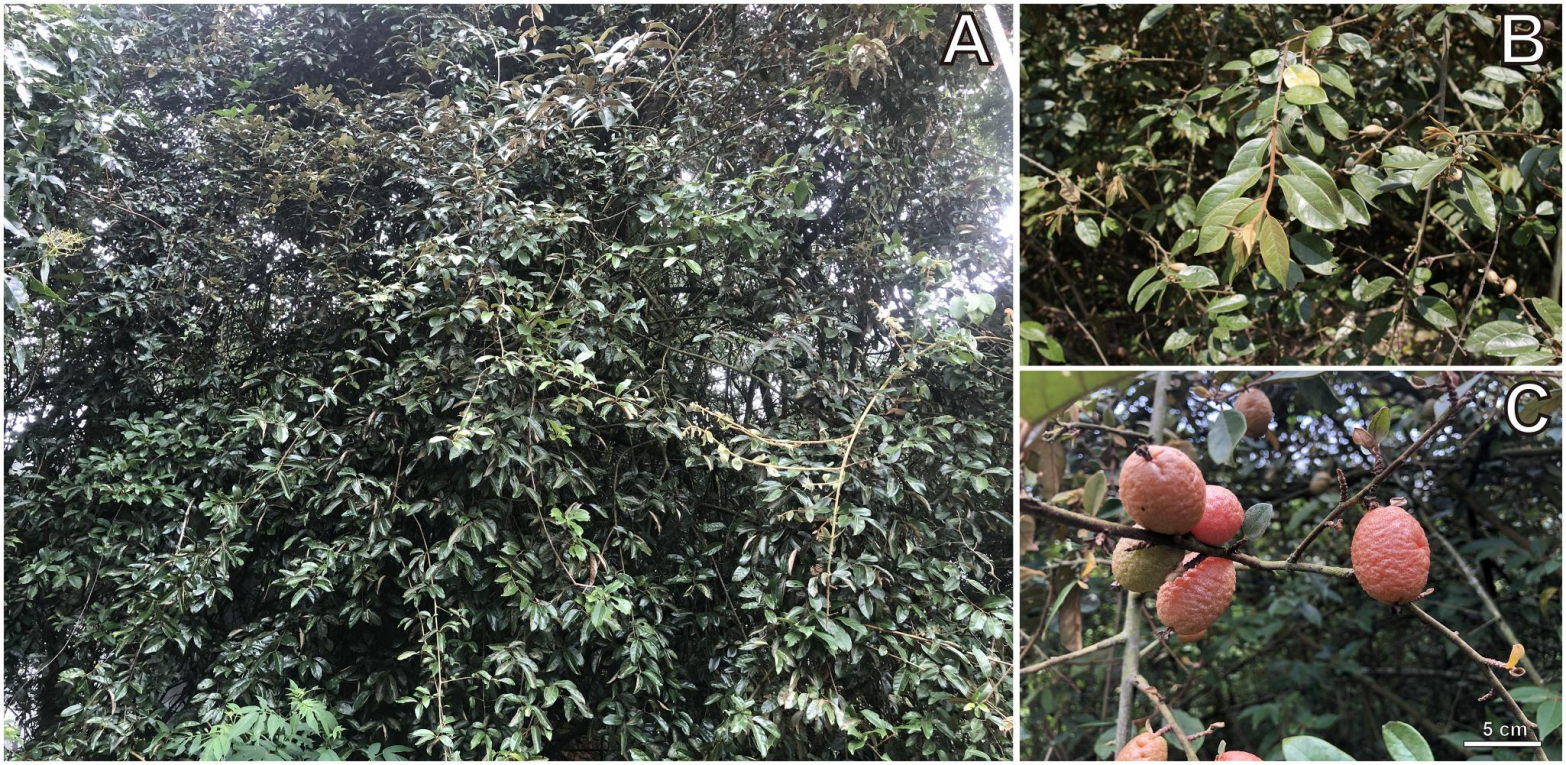
The analyzed sample of *Elaeagnus bambusetorum.* These photos was photographed by Jun Li at Pingbian, Yunnan Province, China. *Elaeagnus bambusetorum* is deciduous or semi-evergreen shrub, whole plant covered with rust-brown scales. The drupe is red at maturity and the seed are elliptic, 8-ribbed. (A) Whole plant. (B) Branches and leaves. (C) Fruits.

Sample leaves of *E. bambusetorum* (Figure 1) were collected from Pingbian, Yunnan Province, China (103°32′34.74″ E, 23°03′26.69″ N). The herbarium specimen was identified and collected by the author, and the voucher specimen was deposited in the Museum of the Beijing Forestry University, China (https://bjfc.bjfu.edu.cn/, Liangcheng Zhao, lczhao@bjfu.edu.cn) under the accession number ENC867090. Total genomic DNA was extracted using the mCTAB protocol (Li et al. 2013). The total DNA was fragmented randomly with an ultrasonicator to construct a 350-bp insert library following the manufacturer’s manual (Illumina Inc., San Diego, CA, USA). The library was sequenced using the Illumina NovaSeq 6000 platform (Novogene, Tianjin, China), yielding approximately 5 GB of data. Trimmomatic v0.36 (Bolger et al. 2014) was used to cut and remove the adaptors and low-quality reads. The chloroplast genome was assembled using the GetOrganelle pipeline (Jin et al. 2020), and genome annotation was performed using the perl script Plann (Huang and Cronk 2015). The annotated genomic sequence was submitted to GenBank with the accession number OR900146. A chloroplast genome map of *E. bambusetorum* was drawn using CPGview (Liu et al. 2023).

The maximum likelihood (ML) method was used to infer the phylogenetic relationships within *Elaeagnus*. All published chloroplast genomes of *Elaeagnus* were downloaded from GenBank. All sequences were aligned with MAFFT v.7 (Katoh and Standley 2013), and TrimAI version 1.3 was used to trim ambiguous alignments. Best-fitting models of nucleotide substitutions were selected using ModelFinder (Kalyaanamoorthy et al. 2017). ML analyses were performed in RAxML-NG (Kozlov et al. 2019) with 500 bootstrap replicates (BS) under the GTR + G model (Pattengale et al. 2009).

## Results

**Figure 2.**
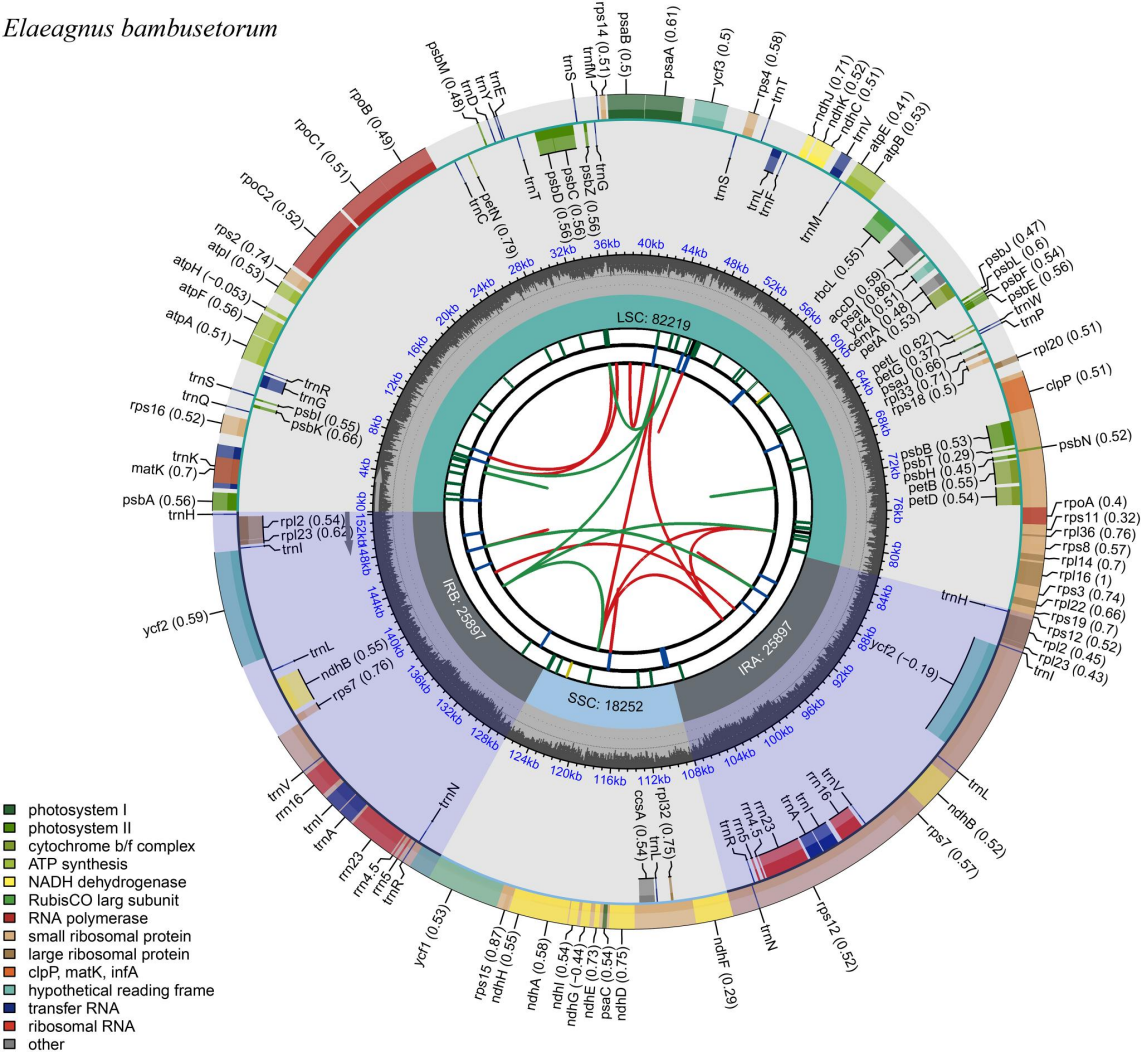
The chloroplast genome map of *Elaeagnus bambusetorum.* From the center going outward, the first circle shows the distribution of the repeats connected with red (the forward direction) and green (the reverse direction) arcs. The second circle displays the tandem repeats marked with short bars. The third circle shows the LSC, SSC, IRa, and IRb regions. The fourth circle shows the percent of GC content. The next circle shows the genes having different colors based on the functional groups. The functional classification is shown at the bottom left. Genes inside the circle are transcribed in a clockwise direction, and those outside are in a counter-clockwise direction.

The complete chloroplast genome of *E. bambusetorum* was 152,265 bp, exhibiting a characteristic circular quadripartite structure. The *E. bambusetorum* chloroplast genome includes a pair of inverted repeats (IRa and IRb: 25,897 bp) and was separated by large single copy (LSC; 82,291 bp) and small single copy (SSC; 18,180 bp) regions. The GC content of the chloroplast DNA was 37.1%. The *E. bambusetorum* chloroplast genome contained 113 unique genes: 79 protein-coding genes, 30 tRNA genes, and four rRNA genes. Among the genes, 45 genes play a role in photosynthesis and 60 genes are associated with self-replication. Ten protein-coding genes, four rRNA genes, and seven tRNA genes were duplicated in the IR regions. A total of 10 protein-coding genes (*atpF*, *ndhA*, *ndhB*, *petB*, *petD*, *rpl2*, *rpl16*, *rpoC1*, *rps16*, and *rps12*) and six tRNA genes (*trnA*-*UGC*, *trnG*-*UCC*, *trnI*-*GAU*, *trnK*-*UUU*, *trnL*-*UAA*, and *trnV*-*UAC*) contained a single intron, whereas two genes (*clpP* and *ycf3*) had two introns (Figure 2, Figures S1–S3).

**Figure 3.**
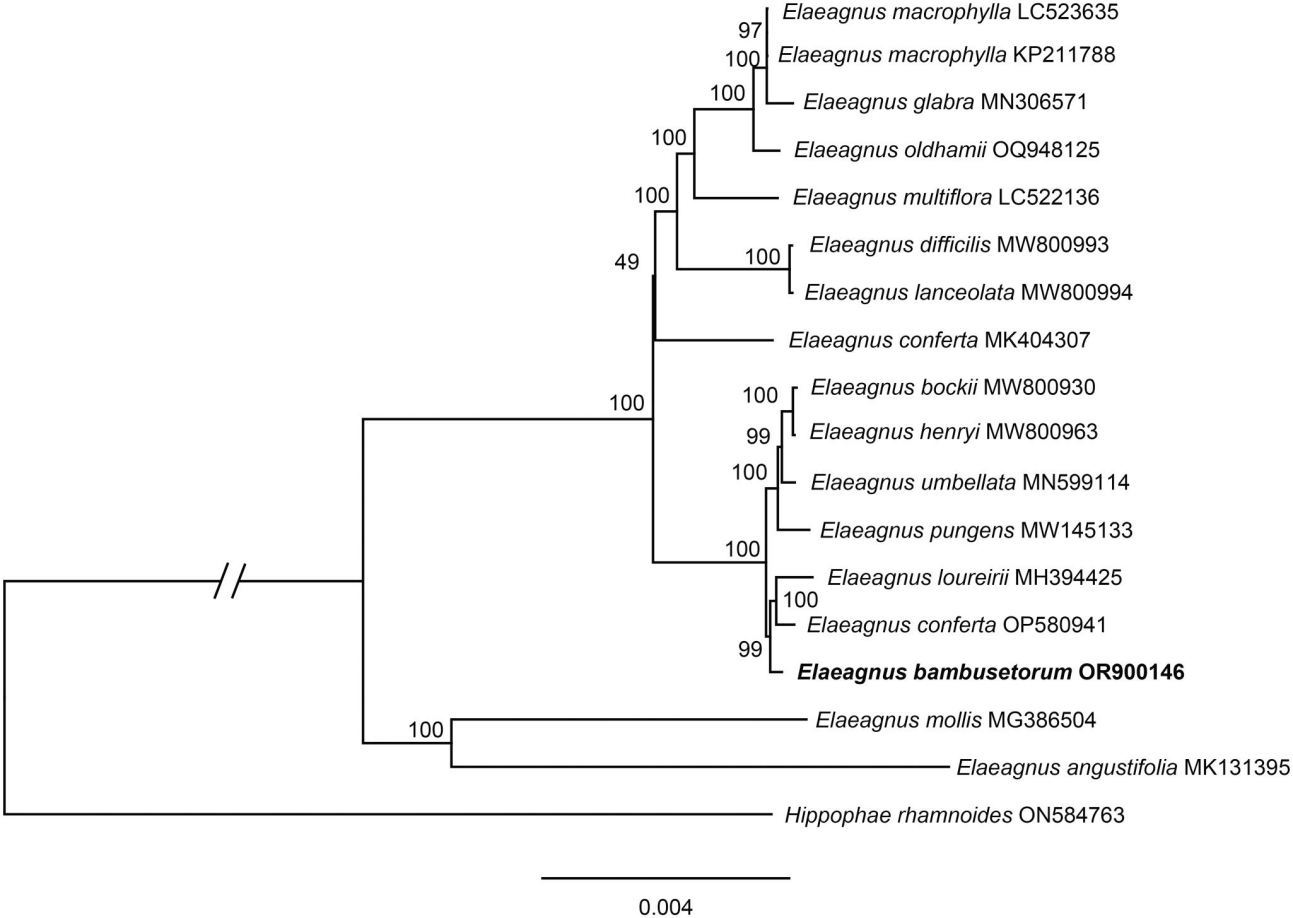
Phylogenetic tree based on whole chloroplasts genome using maximum likelihood (ML) method. The *hippophae rhamnoides* was used as outgroups. Numbers near the branches are bootstrap support (BS) percentages obtained from maximum likelihood inference. The following sequences were used: *E. conferta* MK404307 (Liu et al. 2019), *E. lanceolata* MW800994 (Jin et al. 2022), *E. loureirii* MH394425 (Zeng et al. 2018), *E. pungens* MW145133 (Lu et al. 2022), *E. glabra* MN306571 (Zhao et al. 2020), *E. bockii* MW800930 (Jin et al. 2022), *E. multiflora* LC522136, *E. macrophylla* KP211788 (Choi et al. 2015), *E. henryi* MW800963, *E. angustifolia* MK131395 (Wang et al. 2019), *E. umbellata* MN599114 (Zhao et al. 2020), *E. mollis* MG386504 (Cheng et al. 2020), *E. conferta* OP580941, *E. oldhamii* OQ948125, *E. macrophylla* LC523635, and *E. difficilis* MW800993 (Jin et al. 2022).

The phylogenetic relationship based on the whole chloroplast genome showed that 16 *Elaeagnus* species formed a clade with the highest probability. *Elaeagnus* was divided into two clades with strong support. *E. bambusetorum* was sister to *E. loureirii* and *E. conferta* (Figure 3).

## Discussion and conclusions

In this paper, we successfully sequenced and annotated the complete chloroplast genome of the rare plant *Elaeagnus bambusetorum*. The structure of the genome was found to be consistent with the chloroplast genomes of other *Elaeagnus* species (Choi et al. 2015; Liu et al. 2019; Kim et al. 2020). Phylogenetic analysis based on the chloroplast genome of *E. bambusetorum* supported the division of the genus *Elaeagnus* into two clades, with *E. bambusetorum* forming a sister relationship to *E. loureirii* and *E. conferta***.** Thus, the complete chloroplast genome of *E. bambusetorum* provides valuable genetic information that will aid in species identification, phylogenetic analysis, and conservation efforts within the genus *Elaeagnus*.

## Supplementary Material

Figure S2.jpeg

Figure S3 Sequencing depth and coverage map.jpeg

Figure S1.jpeg

## Data Availability

The genome sequence data are openly available in GenBank of NCBI at (https://www.ncbi.nlm.nih.gov/) under the accession no. OR900146. The associated BioProject, SRA, and Bio-Sample numbers are PRJNA1064668, SRR27547630 and SAMN39439409 respectively.
