# Supplementary figures and images for: The complete chloroplast genome of Elaeagnus bambusetorum hand.-mazz. 1933 and its implications for phylogenetic relationships in the Elaeagnus genus
Source: Mitochondrial DNA B Resour. 2024 Sep 12;9(9):1213–7. doi: 10.1080/23802359.2024.2403413 (PMC11404369; doi:10.1080/23802359.2024.2403413)

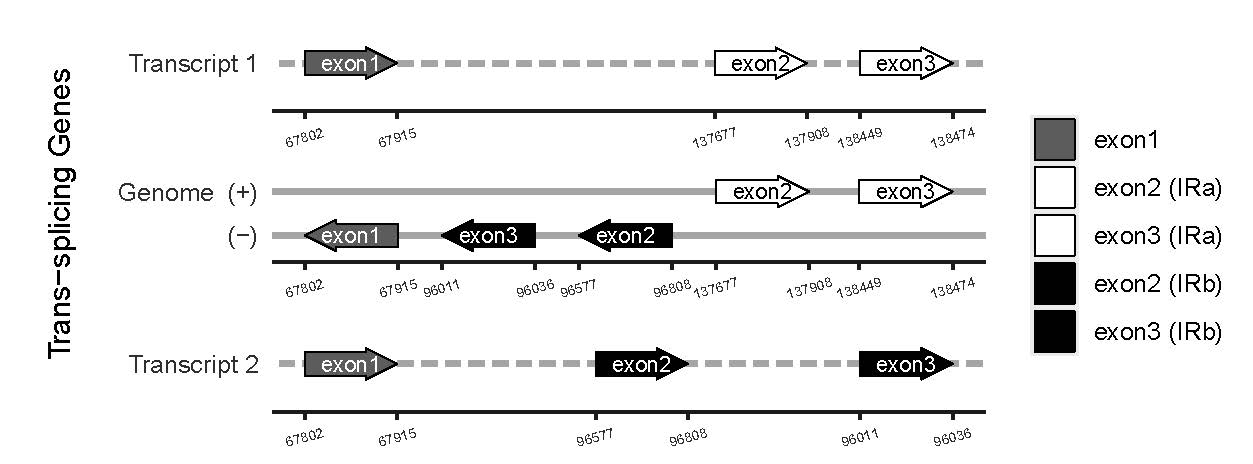

Supplement: Figure S2.jpeg [file TMDN_A_2403413_SM0211.jpeg]

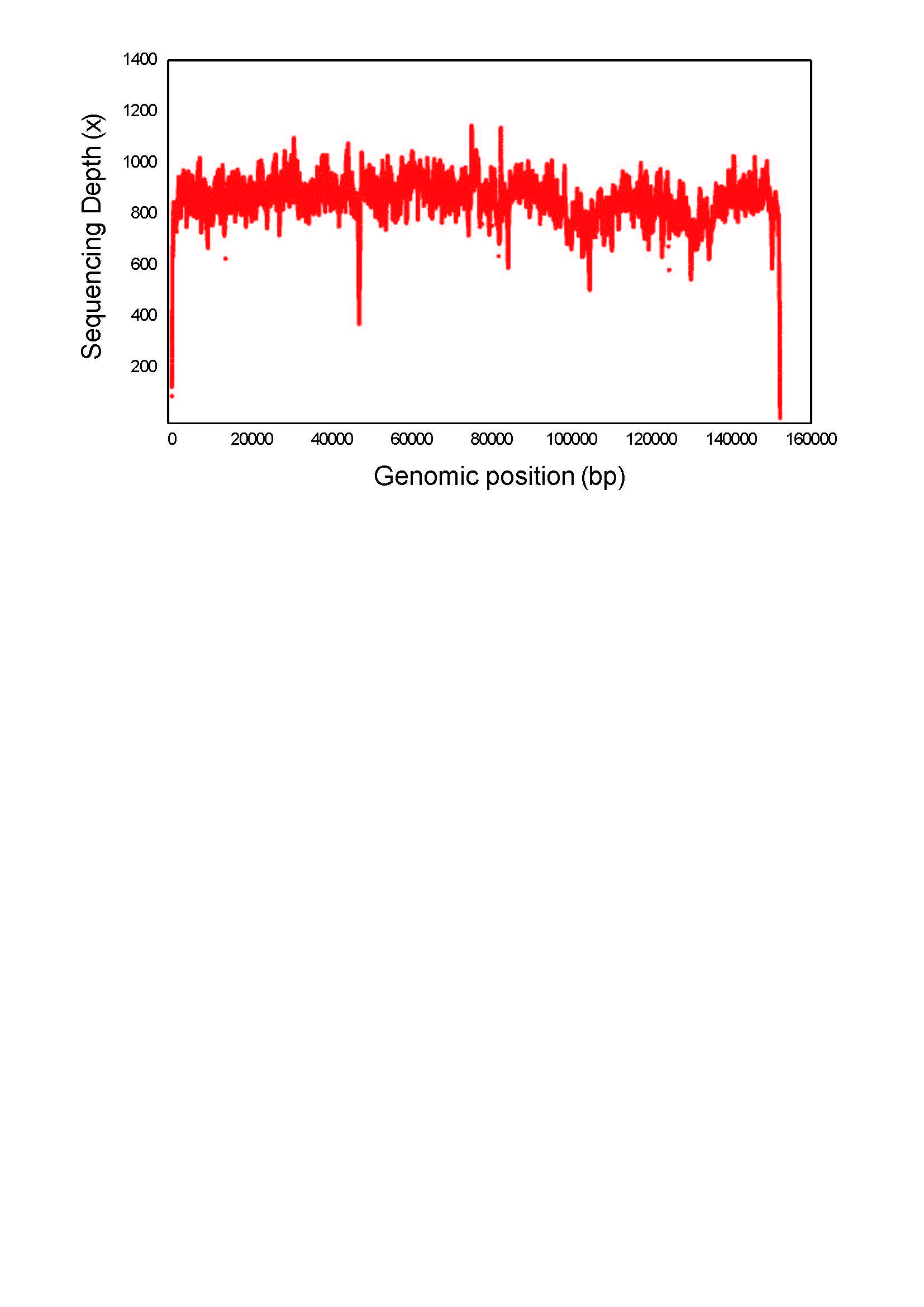

Supplement: Figure S3 Sequencing depth and coverage map.jpeg [file TMDN_A_2403413_SM0210.jpeg]

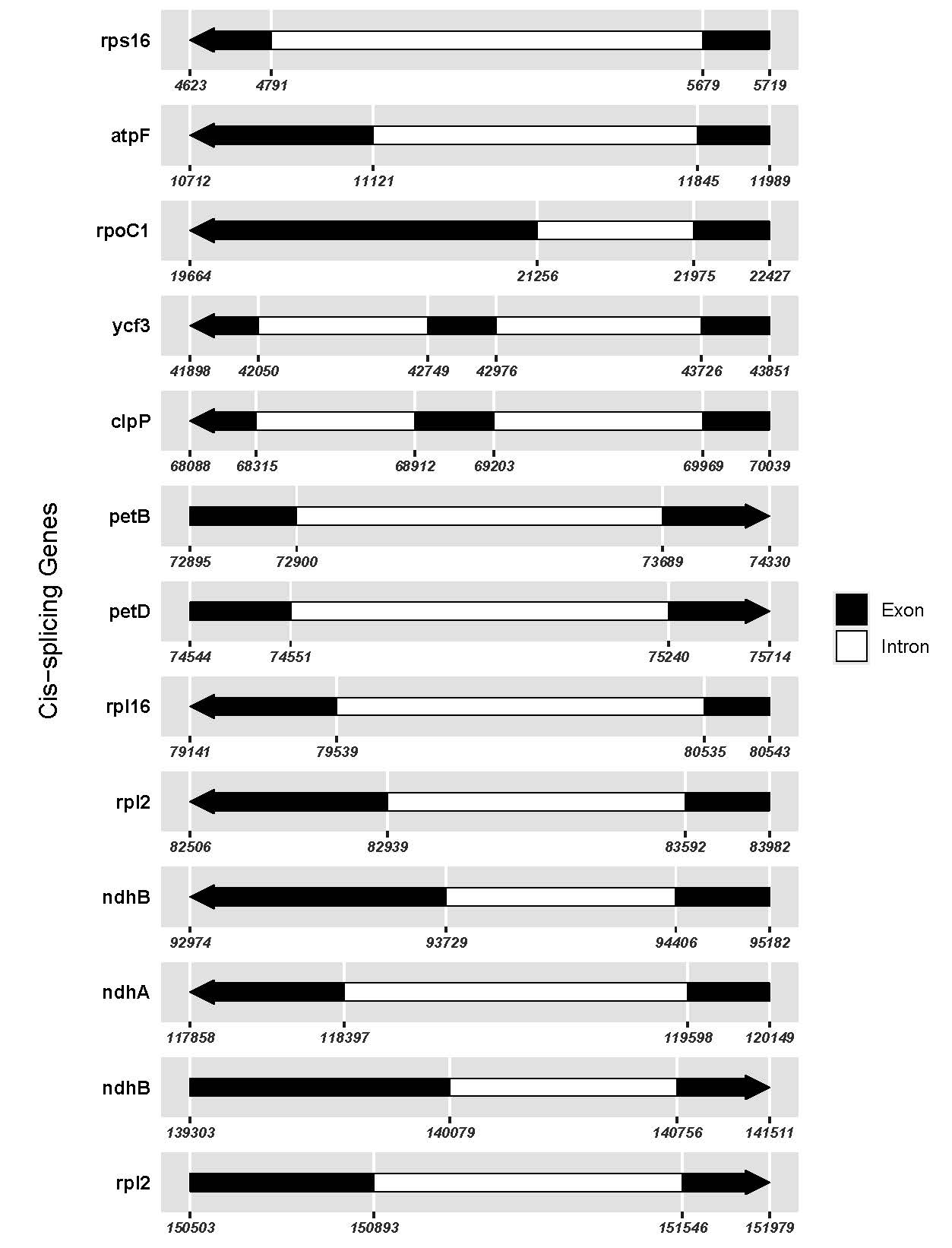

Supplement: Figure S1.jpeg [file TMDN_A_2403413_SM0208.jpeg]
